# Supplementary figures and images for: Recent Divergence and Microgeographic Genetic Structure in an Endangered Australian Songbird: The Southern Black‐Throated Finch
Source: Ecol Evol. 2025 Oct 29;15(10):e72313. doi: 10.1002/ece3.72313 (PMC12570366; doi:10.1002/ece3.72313)

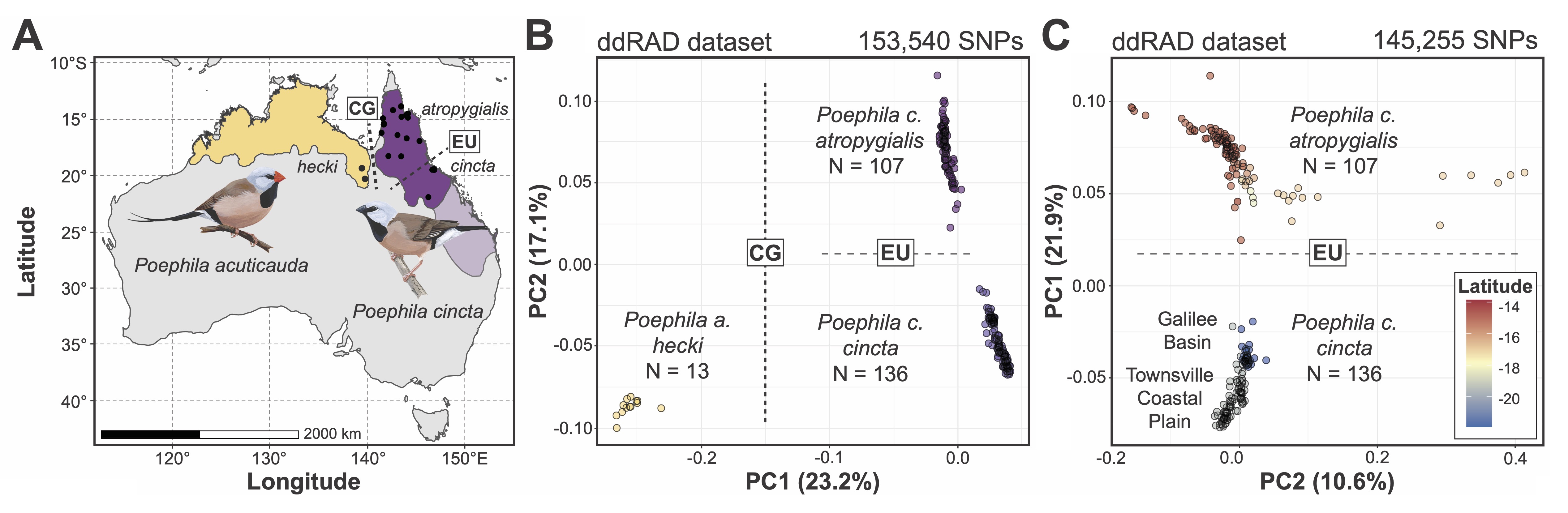

Supplement: Supplementary file 1 — Figure S1: Geographic distribution and genomic divergence between the long‐tailed finch ( Poephila acuticauda hecki ) and both black‐throated finch ( Poephila cincta cincta and P. c. atropygialis) subspecies. (A) Estimated range distribution of the long‐tailed finch (yellow) and black‐throated finch (purple). Recently extirpated regions of the black‐throated finch geographic range (subspecies cincta) are represented with a lighter shade of purple. The geographic locations of populations sampled in this study are represented as black circles (see Table S3 for precise locations). The dashed lines represent the approximate geographic locations of two relevant biogeographic barriers: the Carpentarian Gap (CG) separating the long‐tailed finch (west of barrier) and black‐throated finch (east of barrier) and the Einasleigh Uplands (EU) separating black‐throated finch subspecies atropygialis (north of barrier) from cincta (south of barrier). (B) Principal components analysis based on SNP variation using all ddRAD dataset samples from both species (N = 256) and (C) only samples from the black‐throated finch (N = 243). Biogeographic barriers CG and EU are depicted as labeled dashed lines separating samples from either side of them. Samples are color‐coded by taxon in (B) and by the latitude (i.e., LAT) of the population they were sourced from in (C). See Figure 1 for results with WGS dataset. [file ECE3-15-e72313-s004.jpg]

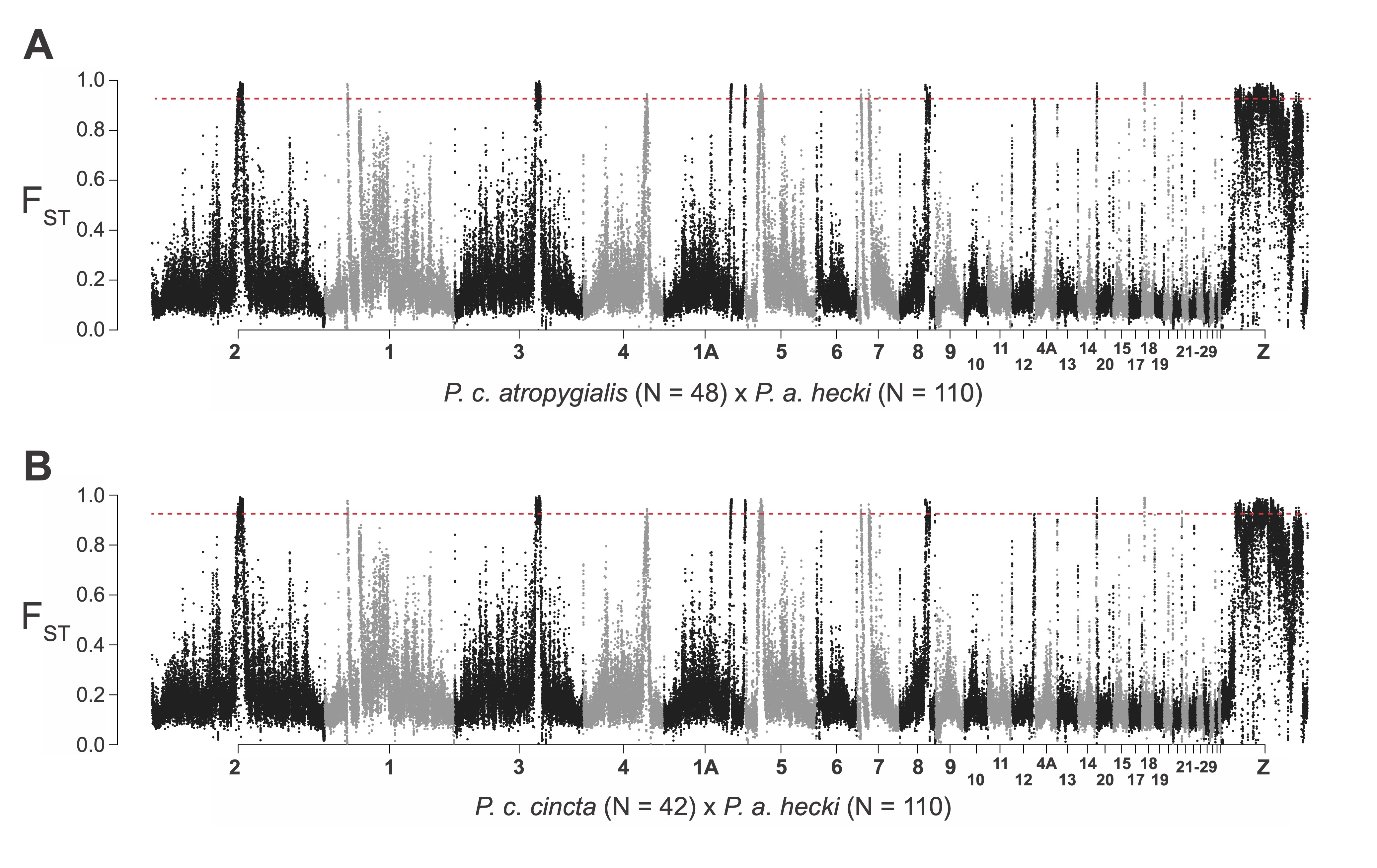

Supplement: Supplementary file 2 — Figure S2: Genomic differentiation (F ST) between the long‐tailed finch (N = 110) and black‐throated finch subspecies (A) atropygialis (N = 48) and (B) cincta (N = 42) in 20 kb windows with 10 kb steps. The dashed red horizontal line represents the 99th percentile threshold for the window based F ST distribution (atr‐hec, F ST = 0.925; cin‐hec, F ST = 0.924). [file ECE3-15-e72313-s001.jpg]

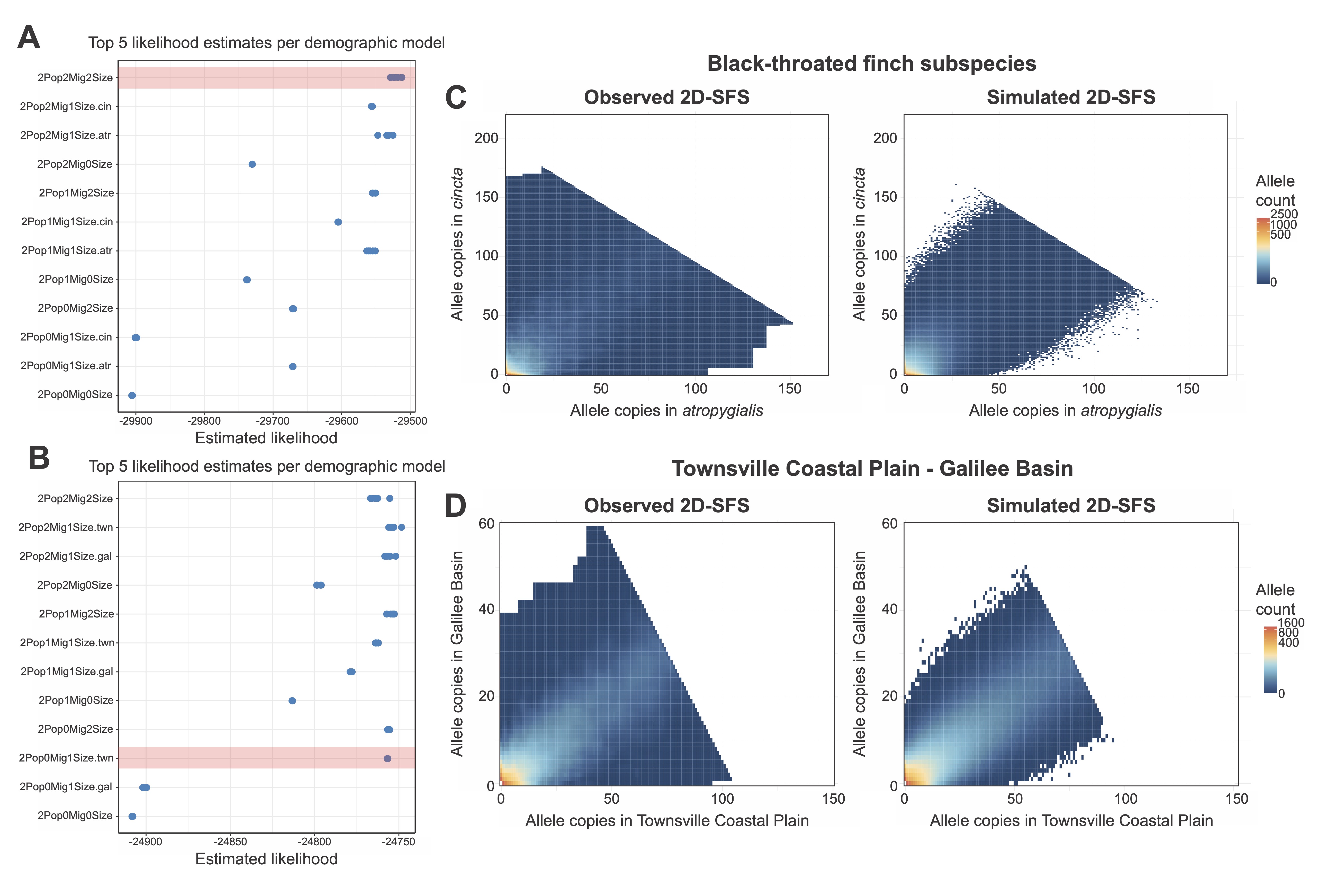

Supplement: Supplementary file 3 — Figure S3: Demographic model inference. (A and B) Top five maximum likelihood scores plotted for 12 competing models, extracted from a set of 100 independent parameter searches per model. The red highlighted box indicates the best‐fitting model based on the Akaike Information Criterion. Comparisons of the observed and simulated two‐dimensional site frequency spectrum (2D‐SFS) under the top‐scoring model for (C) black‐throated finch subspecies atropygialis and cincta (i.e., 2Pop2Mig2Size) and (D) between populations of southern black‐throated finch subspecies cincta from the Townsville Coastal Plain and the Galilee Basin (2Pop0Mig1Size.twn). See Tables S13 and S14 for parameter estimates under the best‐fitting iteration of each model. [file ECE3-15-e72313-s003.jpg]
